# Outcomes of Salvage Autologous Versus Allogeneic Hematopoietic Cell Transplantation for Relapsed Multiple Myeloma After Initial Autologous Hematopoietic Cell Transplantation

**DOI:** 10.4021/jocmr1274w

**Published:** 2013-04-23

**Authors:** Baldeep Wirk, Michael Byrne, Yunfeng Dai, Jan S. Moreb

**Affiliations:** a Division of Hematology-Oncology, University of Florida, USA; b Division of Biostatistics, University of Florida, USA

**Keywords:** Multiple myeloma, Relapse, Salvage hematopoietic cell transplantation

## Abstract

**Background:**

Standard therapy for multiple myeloma (MM) includes initial autologous hematopoietic cell transplantation (autoHCT1) but this is not curative and most patients will relapse. Role of salvage autoHCT2 or allogeneic HCT (alloHCT2) is undefined.

**Methods:**

MM patients who relapsed after autoHCT1 and had salvage autoHCT2 (N = 27) or alloHCT2 (N = 19) between 1995 - 2011 at our institution were studied retrospectively.

**Results:**

Complete and very good partial remission (CR/VGPR) improved from 7% to 56% after autoHCT2 and from 26% to 37% after alloHCT2. Nonrelapse mortality (NRM) at 3 years was 3.7% for autoHCT2 and 5.3% for alloHCT2 (P = 0.901). Median progression free survival (PFS) and overall survival (OS) for autoHCT2 (19 months, 23 months) and alloHCT2 (6 months, 19 months) were not significantly different. On multivariate analysis, time from autoHCT1 to relapse ≥ 1year (HR 24.81, 95% CI 2.4 - 249.9) and maintenance therapy after autoHCT2 (HR 12.19, 95% CI 2.5 - 249.9) impacted OS after autoHCT2. Time from autoHCT1 to relapse < 1 year vs. ≥ 1 year (HR 18.55, 95% CI 2.28 - 150.57) impacted PFS after autoHCT2. For alloHCT2, no factors impacted NRM, PFS or OS. For those with relapse from autoHCT1 < 1 year vs. ≥ 1 year undergoing autoHCT2, median OS was 15 months (range, 1 - 53) vs. not yet reached at 143 months and median PFS was 5 months (range, 1 - 49) vs. not yet reached at 88 months.

**Conclusions:**

Salvage autoHCT2 and alloHCT2 are both feasible for post autoHCT1 MM relapse. Relapse ≥ 1 year from autoHCT1 predicts for better PFS and OS after autoHCT2. Maintenance therapy after autoHCT2 is beneficial.

## Introduction

Standard therapy for multiple myeloma includes initial autologous hematopoietic cell transplantation (autoHCT1) which has been shown to improve the complete remission rate (CR), progression free survival (PFS) and overall survival (OS) compared to standard chemotherapy [1, 2]. The use of novel agents in induction therapy for multiple myeloma followed by consolidation with high dose melphalan and autoHCT and maintenance therapy with lenalidomide has resulted in improvement in the PFS to 46 months compared to 27 months without maintenance therapy [3]. However autoHCT is not curative and most if not all patients will relapse and there is no plateau on the survival curve [4]. Multiple myeloma with adverse cytogenetics such as t(4;14) has only an 8 month median time to progression after autoHCT1; however adverse cytogenetics can be overcome by allogeneic HCT [5, 6]. Data on salvage autologous HCT (autoHCT2) or allogeneic HCT (alloHCT2) are limited and the optimal salvage strategy is unknown.

Allogeneic HCT can lower the risk of recurrence due to the graft versus (vs.) myeloma (GVM) effect mediated by donor T lymphocytes as shown by durable disease remissions induced by donor lymphocyte infusions [7]. Potential benefits of allogeneic HCT have been offset by the high treatment related mortality (TRM) in the past compared to autologous HCT. However, the TRM at 5 years after myeloablative and reduced intensity conditioned allogeneic HCT has decreased over time (48% TRM between 1995 - 2000 vs. 29% TRM between 2001 - 2005) with further improvements expected since this analysis due to improvements in supportive care and greater use of reduced intensity conditioning regimens [8]. The recent introduction of novel agents (thalidomide in 1999, bortezomib in 2003, and lenalidomide in 2006) could also increase cytoreduction and depth of response before allogeneic HCT and thereby result in less TRM.

The aim of this study was to analyze the outcomes of salvage alloHCT2 and autoHCT2 performed after relapse from initial autoHCT1 for MM patients treated in our institution.

## Materials and Methods

This study is a retrospective chart review of multiple myeloma patients > 18 years of age who relapsed after initial autoHCT1 and underwent salvage autoHCT2 or alloHCT2 between January 1995 and December 2011 at our institution. Salvage HCT in this study will be defined as allogeneic or autologous HCT performed at any time after relapse from initial autoHCT1. Tandem auto-auto HCT or auto-alloHCT was excluded. The study was approved by the Institutional Review Board at the University of Florida.

The diagnosis and response criteria of MM were made according to the International Myeloma Working Group (IMWG) criteria [9]. Responses were assessed within 30 days before HCT2 and at day + 100 after HCT2. Cytogenetics were examined at relapse from autoHCT1 and defined as standard risk (hyperdiploidy, t(11;14), t(6;14)), intermediate risk (t(4;14), deletion 13, hypodiploidy by conventional karyotyping) or high risk (17p deletion, t(14;16), t(14;20)) [10]. Karnofsky performance status (KPS) and the HCT specific comorbidity index (CI) were calculated for each patient before HCT2 [11, 12].

### Salvage autologous hematopoietic cell transplantation

Preparative regimens for autoHCT2 were melphalan (Mel) 200 mg/m^2^ (16 patients) or IV busulfan (Bu) 0.8 mg/kg/dose every 6 hours × 16 doses, cyclophosphamide (Cy) 60 mg/kg IV daily × 2 days (9 patients) with 2 patients under 65 years of age also receiving Bu and Cy with etoposide 10 mg/kg IV daily for 3 days. The source of hematopoietic stem cells was from the cryopreserved filgrastim (G-CSF) mobilized peripheral blood stem collection before autoHCT1 in all 27 patients. Infection prophylaxis included levofloxacin, fluconazole and valacyclovir. G-CSF 5 micrograms/kg subcutaneously was administered from day + 6 after stem cell infusion until the absolute neutrophil count (ANC) was > 0.5 × 10^9^/L for 3 consecutive days. All blood products were leukopoor and irradiated. After neutrophil recovery, prophylactic doses of trimethoprim-sulfamethaxazole for 6 months and valacyclovir for 1 year were given.

### Salvage allogeneic hematopoietic cell transplantation

There were 13 matched sibling related donors (6/6) defined by high resolution typing of human leukocyte antigen (HLA) at 6 loci, HLA A, B, DRB1. Five patients had matched (10/10) unrelated donors defined as high resolution molecular HLA typing at 10 loci, HLA A, B, C, DRB1, DQB1. One patient had a haploidentical (6/12) related bone marrow transplant, defined by high resolution molecular HLA typing at 12 loci, HLA A, B, C, DRB1, DQB1, DQA1.

Different published preparative regimens were used for these patients including reduced intensity conditioning regimens in 16 patients. Specifically, 12 patients had fludarabine (Fludara) 30 mg/m^2^ IV daily × 5 days, Bu 0.8 mg/kg/dose IV every 6 hours × 8 doses and rabbit antithymocyte globulin (ATG) 1.5 mg/kg/day IV × 4 days. Two patients had Fludara 30 mg/m^2^ IV daily × 4 days, Mel 140 mg/m^2^ IV × 1 dose, with rabbit ATG 3 mg/m^2^ IV daily × 4 days due to having matched unrelated donors and 1 patient with a matched related donor had Fludara and Mel. The patient with the haploidentical related donor had Fludara 30 mg/m^2^ IV daily × 5 days, Cy 14.5 mg/kg/day IV × 2 days, total body irradiation (TBI) 200 cGy in one fraction. Myeloablative conditionining was with Busulfan 0.8 mg/kg/dose IV every 6 hours × 16 doses, Cy 60 mg/kg daily IV × 2 days (2 patients with matched related donors) and 1 patient with a matched unrelated donor had Bu and Cy with the addition of rabbit ATG 3 mg/kg IV daily × 3 days. G-CSF 5 micrograms/kg daily subcutaneously beginning day + 5 after stem cell infusion until ANC > 1 × 10^9^/L for 3 consecutive days was used in the patient with a haploidentical related donor.

Graft vs. host disease (GVHD) prophylaxis was FK506 (tacrolimus) 0.03 mg/kg IV beginning day -3 with methotrexate 5 mg/m^2^ IV on days + 1, + 3, + 6, + 11 (6 patients), tacrolimus at 0.06 mg/kg po every 12 hours beginning day -3 (12 patients), and tacrolimus 1 mg po bid and mycophenolate mofetil 15 mg/kg po tid and Cy 50 mg/kg/day iv × 2 days on day + 3 and day + 4 after stem cell infusion (1 patient with a haploidentical donor). Generally, the dose of tacrolimus was tapered between day + 60 and day + 100 if there were no signs of GVHD. Acute and chronic GVHD were graded according to international criteria [13, 14].

Supportive and prophylactic antibiotics were used as described above for autoHCT. In addition, CMV PCR weekly monitoring and preemptive therapy were used for all alloHCT2 patients.

### Statistical analysis

Non-relapse mortality (NRM) was defined as death from any cause during the first 28 days of salvage HCT2 or death without evidence of progressive disease with relapse as a competing risk. Relapse was defined as progressive disease after HCT2. For PFS, patients were considered a treatment failure at the time of progression/relapse or death from any cause. For relapse, NRM, PFS, patients alive without evidence of disease relapse or progression were censored at last followup (December 2011). Overall survival (OS) was defined as death from any cause after HCT2. Surviving patients were censored at the time of last contact regardless of ongoing treatment.

OS and PFS were described using the Kaplan-Meier method. The cumulative incidence method was used to estimate relapse and NRM accounting for the presence of competing risks. Prognostic factors for survival were evaluated using the Cox proportional hazards model for univariate analysis. Multivariate analysis included all variables found to be significant at P ≤ 0.10 on univariate analysis. Retention in the step wise model required that the variable be significant at P ≤ 0.05 on multivariate analysis. Analysis was performed using the statistical package SAS version 9.1 (SAS Institute, Cary, NC).

## Results

### Patient characteristics

Patient characteristics of those undergoing salvage autoHCT2 (N = 27) and alloHCT2 (N = 19) are listed in Table 1 and 2. Median followup of both groups was 57 months from diagnosis of MM. All patients except one in the alloHCT2 group had a measurable serum or urine paraprotein level. All patients had reinduction chemotherapy for progressive disease after autoHCT1. Patient characteristics of autoHCT2 and alloHCT2 were not significantly different with respect to gender, stage, high/intermediate risk cytogenetics, immunoglobulin type, beta-2 microglobulin (B2M) at salvage HCT, HCT comorbidity index (CI), lines of chemotherapy before salvage HCT2, chemosensitivity before salvage HCT2, time from diagnosis of MM to autoHCT1, time from autoHCT1 to relapse, time from autoHCT1 to salvage HCT2, salvage HCT2 in first or greater relapse or use of maintenance therapy after HCT2. However, those undergoing alloHCT2 were significantly younger (median age 54 years) than the autoHCT2 group (median age 62 years) (P = 0.002) and had better KPS ≥ 70% (P = 0.031). In addition, more autoHCT2 than alloHCT2 patients had reinduction chemotherapy after relapse from autoHCT1 with novel agents than with conventional agents (P = 0.021).

**Table 1:** Patient Characteristics

Variable	AutoHCT2 N = 27	AlloHCT2 N = 19
Male/Female	16/11	10/9
Median age years (range)*	62 (32 - 69)	54 (43 - 63)
Median months from diagnosis to autoHCT1	8 (3 - 39)	8 (5 - 30)
KPS at HCT2: ≥ 70% vs. <70% *	20/7	19/0
HCT comorbidity index		
0, 1	5	8
2, 3	10	7
> 3	12	4
Durie-Salmon stage :I/II/III/unknown	4/6/17	0/5/13/1
ISS stage: I/II/III/unknown	11/4/5/7	9/5/3/2
B2microglobulin at HCT2: ≥ 3.5/<3.5/unknown	9/14/4	4/14/1
Cytogenetics		
High risk/intermediate	9	4
Standard risk	15	13
Unknown	3	2
IG subtype		
IgG	12	10
IgA	7	8
Light chain	8	0
Nonsecretory		1
Lines of chemo before HCT2	1 (1 - 5)	2 (1 - 5)
Chemosensitive before HCT2:Yes/No	11/16	11/8
Induction before HCT2*		
Conventional chemo	6	12
Novel agents	21	7
Time from autoHCT1 to relapse: months (range)	16.5 (4 - 42)	12 (2 - 45)
Time from autoHCT1 to HCT2 months (range)	30 (5 - 104)	21 (7 - 91)
conditioning for autoHCT1	2/7/18	1/12/6
BuCY/BuCyVP16/melphalan		
Conditioning for alloHCT2: Reduced intensity (FLU/BU N = 12, FLU/MEL N = 3, FLU/CY/TBI N = 1), Myeloablative (BU/CY N = 3)	Reduced intensity conditioning 16	
Myeloablative 3		
Conditioning for autoHCT2	9/2/16	
BuCy/ BuCyVP16/melphalan		
Donor type		
Matched sibling related 6/6		13
Matched unrelated 10/10		5
Haploidentical related 7/14	1
Stem cell type BM/PB	0/27	1/18
Year of HCT2*		
1995 - 2005	6	8
2006 - 2011	21	11
Disease status before/after HCT2		
CR	0/4	2/3
VGPR	2/11	3/4
PR	9/7	6/4
SD	1/2	8/0
PD	15/3	0/8
Maintenance after HCT2: yes vs. no	12/15	3/16
Median months of follow up from diagnosis (range)	57 (19 - 115)	57 (22 - 154)

* statistically significant.

**Table 2:** AlloHCT2 Patient Characteristics

Variable	Allohct2 N = 19
Donor/recipient gender	
M/M	6
M/F	8
F/F	2
F/M	3
Conditioning for alloHCT2
Reduced intensity conditioning
FLU/BU	12
FLU/MEL	3
FLU/CY/TBI	1
Myeloablative BU/CY	3
GVHD prophylaxis	
FK	11
FK/MTX	7
CSA/MMF	1
DLI use, yes/no	10/9
ATG use, yes/no	15/4
Acute GVHD	
None	6
I-II	6
III-IV	7
Chronic GVHD	
None	12
Limited	2
Extensive	5
Causes of death	
PD	5
GVHD	3
Infection	3
Renal failure	1

With regards to the cytogenetics for autoHCT2 group, 15 patients were standard risk, 8 intermediate risk (7 had deletion 13, 1 with t(4;14)) and 1 patient was high risk with deletion 17p. In the alloHCT2 group, 13 patients were standard risk, 1 intermediate risk with deletion 13, and 3 patients were high risk with deletion 17p.

The median interval from autoHCT1 to autoHCT2 was 30 months and from autoHCT1 to alloHCT2, 21 months. The median time from autoHCT1 to relapse in the autoHCT2 and alloHCT2 group was 16.5 months and 12 months, respectively, and was not statistically different. Numbers of salvage HCT2 increased after 2006 (Table 1). Significantly more autoHCT2 were done after 2006 than alloHCT2 (P = 0.033).

Only 10.5% (2/19) in the alloHCT2 group and 14.8% (4/27) in the autoHCT2 group underwent salvage HCT2 < 1year from autoHCT1 and this was not statistically different in the two groups (P = 1.000). Furthermore, 47.4% (9/19) in the alloHCT2 group and 44.4% (12/27) in the autoHCT2 group relapsed < 1 year from autoHCT1 (P = 0.845). After autoHCT2, 12/27 patients received maintenance therapy (3 were given thalidomide, 3 lenalidomide, 3 bortezomib, 3 Cy). Post alloHCT2, 3 patients received Cy maintenance with the remainder (16) receiving no maintenance therapy.

### Disease response and survival

The CR/VGPR improved from 7% to 56% after autoHCT2 and from 26% to 37% after alloHCT2 (Table 1). Of 15 patients with progressive disease (PD) undergoing autoHCT2, 5 achieved CR/VGPR and 7 achieved PR while 3 remained in PD. For those entering salvage autoHCT2 in PD, the 1 year PFS was 50% (range, 23.8-76.2%), 3 year PFS was 41.7% (range, 15.2-68.1%), 1 year OS was 85.7% (range, 67.4-100%) and 3 year OS was 45% (range, 16.1-73.9%).

**Figure 1:**
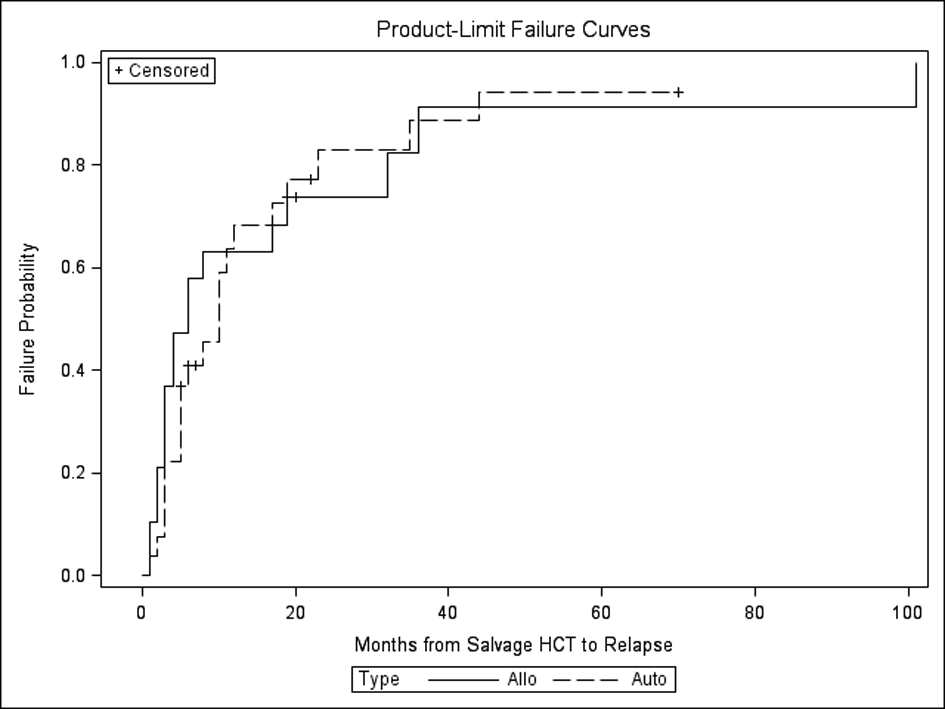
Relapse for salvage autoHCT2 versus alloHCT2, P = 0.605.

**Figure 2:**
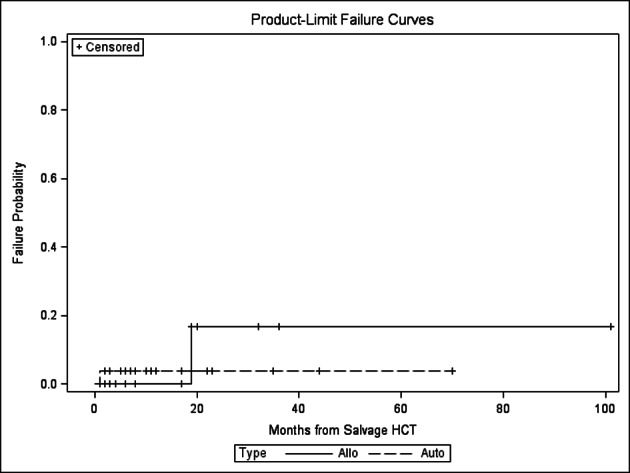
Nonrelapse mortality for salvage autoHCT2 versus alloHCT2, P = 0.901.

**Figure 3:**
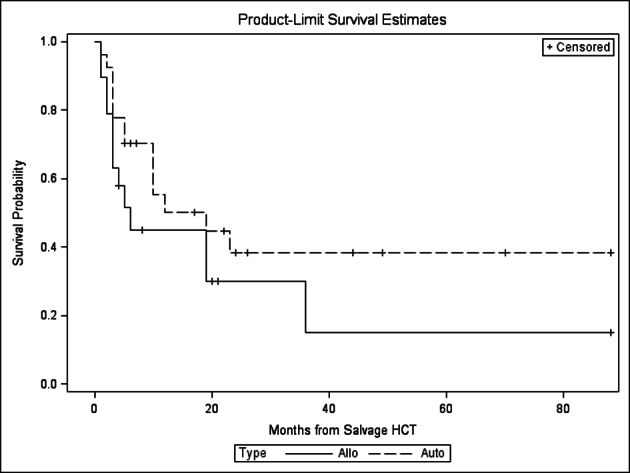
Progression free survival for salvage autoHCT2 and alloHCT2, P = 0.156.

**Figure 4:**
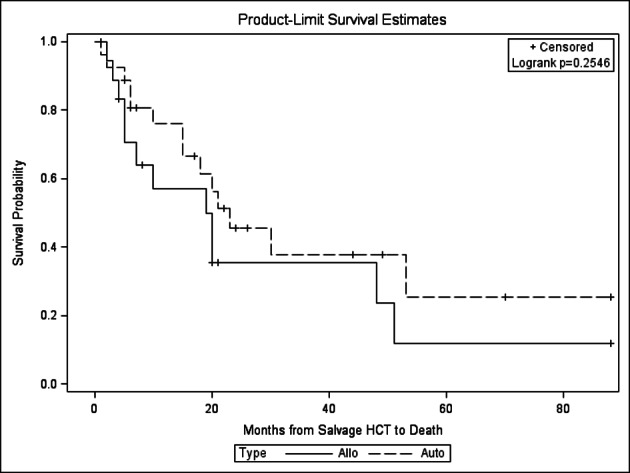
Overall survival for salvage autoHCT2 versus salvage alloHCT2, P = 0.255.

There was no statistically significant difference in relapse, NRM, PFS and OS between the autoHCT2 vs. alloHCT2 group (Fig. 1, 2, 3, 4). The relapse rate at 3 years was 91% (95% CI 76-100%) for alloHCT2 and 88% (95% CI 74-100%) for autoHCT2. Non-relapse mortality (NRM) at 3 years was 3.7% for autoHCT2 and 5.3% for alloHCT2 (P = 0.901). Median PFS and OS for autoHCT2 (19 months, 23 months) and for alloHCT2 (6 months, 19 months) were not significantly different (Fig. 3, 4).

On univariate analysis the following factors were analysed for their impact on OS in the autoHCT2 group: time from autoHCT1 to salvage HCT2 < 1 year vs. ≥ 1 year or < 2 years vs. ≥ 2 years, time from autoHCT1 to relapse < 1 year vs. ≥ 1 year or < 18 months vs. ≥ 18 months, and the following factors at autoHCT2: age, gender, KPS < 70% vs. ≥ 70%, HCT CI < 2 vs. ≥ 2, stage by Durie-Salmon and International Staging System, B2M < 3.5 mg/L vs. ≥ 3.5 mg/L, albumin < 3.5 g/dL vs. ≥ 3.5 g/dL, immunochemical type of MM, induction chemotherapy with conventional vs. novel agents, number of lines of chemotherapy, chemosensitivity vs. chemoresistance, standard vs. intermediate vs. high risk cytogenetics, disease status CR/VGPR vs. others, time from autoHCT1 to relapse, type of relapse bone marrow vs. extramedullary, time from relpase to autoHCT2. Additionally, we analyzed best response after HCT2 CR/VGPR vs. others, time from diagnosis to autoHCT1, conditioning before autoHCT2 melphalan vs. others, stem cell source before HCT2, maintenance therapy after HCT2 none vs. given, autoHCT2 in first or greater relapse, year of HCT2 < 2006 vs. ≥ 2006, time from HCT2 to relapse, and relapse after HCT2 yes vs. no.

The following factors showed significant impact on OS after autoHCT2 on univariate analysis: time from autoHCT1 to relapse < 1 year vs. ≥ 1 year (P = 0.0035) and < 18 months vs. ≥ 18 months (P = 0.0108), HCT CI < 2 vs. ≥ 2 (P = 0.0205), B2M at HCT2 < 3.5 vs. ≥ 3.5 mg/L (P = 0.0248), conditioning before autoHCT2 melphalan vs. others (P = 0.0302), best response after autoHCT2 CR/VGPR vs. others (P = 0.0442), maintenance therapy after autoHCT2 given vs. none (P = 0.04), and time from autoHCT2 to relapse (P = 0.0104).

**Figure 5:**
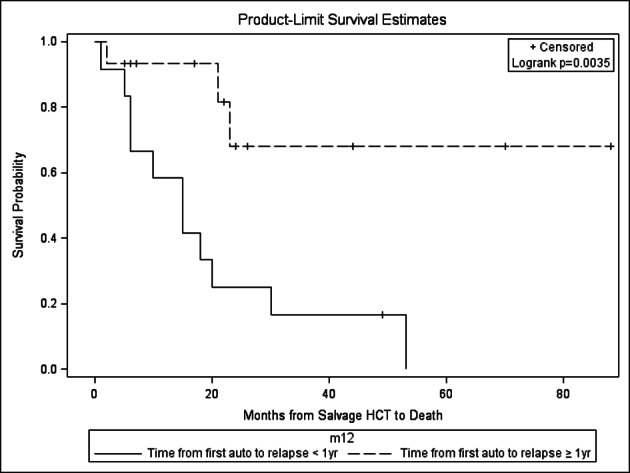
Overall survival for salvage autoHCT2: impact of time from first autoHCT1 to relapse, P = 0.003.

On multivariate analysis, time from autoHCT1 to relapse < 1 year vs. ≥ 1year (HR 24.81, 95% CI 2.4 - 249.9) and no maintenance therapy vs. given after autoHCT2 (HR 12.19, 95% CI 2.5 - 249.9) significantly impacted OS after autoHCT2. Also on multivariate analysis, only time from first autoHCT1 to relapse < 1 year vs. ≥ 1 year (HR 18.55, 95% CI 2.28 - 150.57) impacted PFS after autoHCT2. On the other hand, no factors impacted NRM after autoHCT2. For those with relapse from autoHCT1 < 1 year vs. ≥ 1year undergoing autoHCT2, median OS was 15 months (range, 1 - 53) vs. not yet reached at 143 months and median PFS was 5 months (range, 1 - 49) vs. not yet reached at 88 months (Fig. 5). Relapse was the major cause of death after autoHCT2, with 10 patients dying of PD, 3 from infection and 1 from renal failure.

For the alloHCT2 group, the same variables were tested on univariate analysis for impact on OS as for autoHCT2 with the addition of acute and chronic GVHD, sibling donor vs. unrelated donor, donor/recipient gender female/male vs. other, reduced intensity vs. myeloablative conditioning, ATG use, and donor lymphocyte infusions. Only best response after alloHCT2 (CR/VGPR vs. other) impacted OS after alloHCT2 on univariate analysis (P = 0.0248). On multivariate analysis, no factors were found to impact OS and PFS after alloHCT2 but this maybe attributed to the small sample size. For alloHCT2, OS was similar for patients with autoHCT1 to relapse < 1 year vs. ≥ 1 year (P = 0.214). The median PFS for alloHCT2 with relapse from autoHCT1 < 1year vs. ≥ 1 year was 4 months (range, 1 - 20) and 19 months (range, 1 - 88), respectively, and this was not statistically different (P = 0.354). The median OS for alloHCT2 with relapse from autoHCT1 < 1 year vs. ≥ 1 year was 32 months (range, 17- longest follow up time 115 months) vs. 66 months (range, 19-longest follow up time 143 months) but this was not statistically different (P = 0.214). After alloHCT2, the major cause of death was PD (N = 5) followed by GVHD (N = 3), infection (N = 3) and renal failure (N = 1).

## Discussion

Standard therapy for MM includes consolidation with initial high dose therapy and autoHCT1 which results in improvements in PFS and OS [1, 2]. In a sub analysis of 2 prospective studies, tandem autoHCT improved OS in those who did not achieve at least VGPR after autoHCT1 [15, 16]. Novel agents in induction therapy have led to greater CR rates than conventional chemotherapy agents such vincristine, doxorubicin and dexamethasone (30% vs.10%) and autoHCT1 increases CR by 20%, lessening the need for upfront tandem autoHCT [17]. Current standard practice has shifted now to maintenance therapy with agents such as lenalidomide after autoHCT1 and this has been shown to improve PFS from 26 months with placebo to 46 months with lenalidomide [3]. Similarly, in a study by Attal et al which assessed lenalidomide maintenance after autoHCT1 or tandem autoHCT (performed in those who did not achieve at least VGPR after autoHCT1), the probability of remaining free of disease progression for 4 years was only 43% in the lenalidomide maintenance arm vs. 22% in the placebo group [18]. So despite the use of novel agents in induction and maintenance after autoHCT, the majority of MM patients will relapse and there is no plateau on the survival curve [3, 4, 18]. There is a pressing need to gather data to guide clinical decisions regarding the optimal salvage strategy after MM relapse from initial autoHCT1 since data are limited and come from retrospective studies. There is only one other study in the published literature on salvage autoHCT2 vs. alloHCT2 after relapse from autoHCT1 (Table 3) [19]. The 2 other studies on salvage autoHCT2 vs. alloHCT2 included patients who relapsed after upfront tandem autoHCT or also included patients who had tandem autoHCT or tandem auto-alloHCT performed if they were in partial remission after the initial autoHCT1 and so are not directly applicable to the present discussion of the large majority of patients in this era with relapse after single autoHCT1 [20, 21]. The remaining studies have been a separate exploration of either salvage autoHCT2 or salvage alloHCT2 after relapse from autoHCT1 [22-27].

**Table 3:** Studies on Salvage Autohct2 Versus Salvage Allohct2 After Relapse From Initial Autohct1

Variable	Qazilbash et al	This study
Year inclusive	1992 - 2006	1995 - 2011
# of patients autoHCT2/alloHCT2	14/26	27/19
Time from AutoHCT1 and AutoHCT2 (months)	25	30
Time from AutoHCT1 to AlloHCT2 (months)	17	21
Disease response post autoHCT2, CR/VGPR/PR	21%/-/43%	15%/41%/26%
Disease response post alloHCT2, CR/VGPR/PR	31%/-/38%	16%/21%/21%
NRM after autoHCT2/alloHCT2	7%/11%	3.7%/5.3%
Median PFS post autoHCT2/alloHCT2 (months)	6.8/7.3	19/6
Median OS post autoHCT2/alloHCT2 (months)	29.5/13	23/19
Prognostic Factors	Univariate analysis: time from autoHCT1 to alloHCT2 > 1 year (P = 0.02) predicted significantly better OS for alloHCT2 No factors impacted OS in autoHCT2 group	Multivariate analysis: relapse from autoHCT1 ≥ 1 year favorably impacted PFS and OS after autoHCT2. Also, maintenance therapy after autoHCT2 favorably impacted OS after autoHCT2. No factors impacted PFS/OS after alloHCT2.

In this study, chemosensitivity before salvage autoHCT2 was not found to impact PFS or OS. In fact of 15 patients with PD, 5 achieved CR/VGPR and 7 PR after autoHCT2. This is in comparison to other studies that suggested autoHCT not to be effective in resistant relapsed patients, none of whom attained CR and all of whom had early mortality [28]. Best response (CR/VGPR) was 56% after autoHCT2 and 37% after alloHCT2 and this difference reached statistical significance (P = 0.008) and it impacted OS on univariate analysis but not on multivariate analysis for either autoHCT2 or alloHCT2.

In our study, those with a year or more from autoHCT1 to relapse had the most favorable outcomes after autoHCT2 with respect to PFS and OS (Fig. 5). This is in comparison to other studies which have variably shown duration from autoHCT1 to relapse of > 9 months vs. > 18 months vs. > 24 months vs. ≥ 36 months as having a favorable impact on OS [22-25]. Still other studies have not shown an effect of response duration after autoHCT1 on outcomes after salvage autoHCT2 [19]. Regardless, these results are better than chemotherapy alone, as shown in a study comparing autoHCT2 to salvage chemotherapy for relapse of MM ≥ 18 months after autoHCT1 with median OS of 3.9 years vs. 1.8 years (P = 0.0011) [22].

In comparison to other published studies, our study is the first to show maintenance therapy after salvage autoHCT2 has beneficial effects on OS [24]. Only 3 patients received maintenance therapy after alloHCT2, so firm conclusions cannot be made as to any potential benefits. Indeed, there were no prognostic factors impacting relapse, NRM, PFS or OS after alloHCT2. This may be due to the drawbacks of most retrospective single center studies, namely a small sample size with patients accrued over 16 years and given heterogeneous therapy. For example, we were not able to confirm results from other salvage alloHCT2 studies showing absence of chronic GVHD, lack of chemosensitivity and high risk cytogenetics impacted OS [26]. Nonetheless these results provide important insights and show the feasibility of both alloHCT2 and autoHCT2 which have similar NRM, PFS, OS. Relapse remained the major cause of death in both autoHCT2 and alloHCT2 that was partly abrogated by maintenance therapy after autoHCT2.

Larger number of patients enrolled in prospective multicenter studies comparing salvage autoHCT2 vs. alloHCT2 vs. chemotherapy with novel agents is needed to detect differences in outcomes and determine the optimal therapeutic strategy. A phase III clinical study (Myeloma X, http://www.ukmf.org.uk/trials.htm) in the United Kingdom is currently enrolling MM patients who relapse ≥ 18 months after autoHCT1 to receive bortezomib, doxorubicin and dexamethasone with randomization thereafter to autoHCT2 vs. maintenance with low dose cyclophosphamide. In the United States, enrollment in prospective trials of salvage autoHCT2 vs. alloHCT2 for MM is limited by the lack of Medicare insurance approval for allogeneic HCT in multiple myeloma.

### Conclusion

Our study highlights the feasibility and usefulness of both salvage autoHCT2 and alloHCT2 after MM relapse from autoHCT1. Relpase ≥ 1 year from autoHCT1 predicts for better PFS and OS in the autoHCT2 group. Those with progressive disease can also be salvaged by autoHCT2. Maintenance therapy after autoHCT2 is beneficial and should routinely be used. Prospective multicenter studies comparing these salvage strategies are urgently needed.
